# Current Perspectives and Effectiveness of Fertility Preservation in Cancer Patients—Data from Serbian National Referral Center

**DOI:** 10.3390/healthcare14203469

**Published:** 2026-10-15

**Authors:** Katarina Stefanovic, Aleksandra Beleslin, Igor Pilic, Ivana Likic Ladjevic, Jelena Stojnic, Jelena Micic, Aleksandar Stefanovic, Aleksandra Didanovic, Anja Stepanovic, Jelena Dotlic

**Affiliations:** 1 Clinic of Obstetrics and Gynecology, University Clinical Centre of Serbia, Dr. Koste Todorovica 26, 11000 Belgrade, Serbia; 2 Medical Faculty, University of Belgrade, Dr. Subotica 8, 11000 Belgrade, Serbia

**Keywords:** oncofertility, Serbia, oncologic outcome, obstetric outcome

## Abstract

**Highlights:**

**What are the main findings?**

• The survival rate for patients with early-stage cervical cancer was 93.3% but attempted pregnancy rate was 26.7%. The survival rate for patients with early-stage endometrial malignancy was 100% but attempted pregnancy rate was 29.9%. The survival rate for patients with stage I borderline ovarian tumors was 100% and the pregnancy rate was 44.8%. The survival rate for patients with non-epithelial ovarian cancers was 87.2%, but we had just seven successful pregnancies.
• Analyzed program-level indicators showed that the effectiveness of our oncofertility program corresponds with general recommendations and data from other centers.

**What are the implications of the main findings?**

• Future efforts should be directed towards an integrated multidisciplinary approach, new guidelines, standardized protocols, long-term follow-up, clear institutional referral, and having national and international registries of oncofertility patients.
• The study comprehensively assessed the effectiveness of the oncofertility program and presented the 10-year experiences with fertility-sparing treatment at the Ob/Gyn Clinic University Clinical Center of Serbia, where fertility-sparing treatments and cryopreservation of reproductive materials are performed within the same institutional framework. The presented successful oncologic and obstetrical results support the feasibility of achieving favorable oncologic and reproductive outcomes in appropriately selected patients by following current treatment protocols.

**Abstract:**

Background/Objectives: Despite numerous fertility-sparing treatments, there are still uncertainties. We present the experience and effectiveness of the oncofertility program from the University Clinical Center of Serbia. Methods: Study included all malignancy patients undergoing fertility-sparing treatment during a 10-year period. For all patients, we registered malignancy type, therapy type, course and duration, the response to therapy, complications and recurrence, and overall survival. All attempted and achieved pregnancies were recorded. We noted all pregnancy complications and gestational week at pregnancy end and calculated live-birth rates. Results: Fifteen patients with early-stage cervical cancer (squamocellular 11; adenocarcinoma 4) had abdominal radical trachelectomy. Outcomes of our patients were excellent, but just three patients delivered healthy children. In total, 87 patients with early-stage endometrial malignancy (atypical hyperplasia 24, intraepithelial neoplasm 24, carcinoma 39) received hormonal therapy. Complete remission was achieved at 74.7% but only nine achieved pregnancy. Moreover, 52 patients with stage I borderline ovarian tumors were treated. The overall survival rate was 100% and the pregnancy rate was 44.8%. We also treated 39 patients with non-epithelial ovarian cancers (sexcord–stromal tumors 22, malignant germ cell tumors 17). The overall survival rate was 87.2%, but we had just seven successful pregnancies. In our institution, oocytes were cryopreserved in 63, embryos in 6, and ovarian tissue in 8 patients, out of which two achieved pregnancy and one had a term delivery of a healthy child. Conclusions: Our findings support the feasibility of favorable oncologic and reproductive outcomes in carefully selected oncofertility patients following current guidelines.

## 1. Introduction

Although malignancy is more commonly diagnosed in older adults, an increasing incidence of various malignant diseases has recently been reported in younger adults [1,2]. According to the World Health Organization, cancer diagnosed during childhood, adolescence, or reproductive age presents a growing clinical and public health challenge. In women younger than 40 years, the most common malignancies are breast cancer, thyroid cancer, cervical cancer, leukemia, and ovarian cancer [3,4]. When young people are diagnosed with cancer, they have often not completed childbearing and must postpone family planning because of the disease [1,2].

Owing to early diagnosis and advances in systemic therapy, surgery, radiotherapy, and targeted therapy, cancer survival rates have improved, and the focus of care has shifted from survival alone toward long-term quality of life [3,5]. However, anticancer treatments, especially chemotherapy and pelvic radiotherapy, are often gonadotoxic and may disrupt endocrine functioning, causing premature ovarian insufficiency, infertility, and/or early menopause [1,6]. Annually, more than 40,000 women of reproductive age face infertility due to different cancer treatments [2,3,4].

Oncofertility is a multidisciplinary field that helps patients to maintain their fertility while undergoing cancer treatment [6,7]. Primarily, it aims to preserve reproductive potential and endocrine function while ensuring oncologic treatment is not compromised. One of the reasons for the rising interest in oncofertility is increased awareness of quality of life in cancer survivors. Parenthood is a well-known aspect of personal well-being. Therefore, a careful individualized approach is needed for each patient [8]. Patients who qualify for fertility-sparing treatment must be informed about the potential impact of cancer treatment on fertility and should be referred to reproductive specialists as early as possible after confirming diagnosis and before beginning therapy [9,10].

Oncofertility bridges the two complementary fields of oncology and reproductive medicine. Its various methods can be performed before, during, or after cancer treatment [11] and are chosen based on tumor histology and stage, treatment type, and individual patient characteristics (age, parity, ovarian reserve, etc.). Established fertility preservation techniques in women are cryopreservation of oocytes, embryos, and ovarian tissue, pharmacological ovarian suppression with gonadotropin-releasing hormone (GnRH) analogs, ovarian and uterine transposition, and different conservative surgeries [6,7,8,9]. If fertility preservation is infeasible or unsuccessful, alternatives should be discussed, such as donor egg, sperm, embryo, and adoption [12].

Fertility-sparing surgeries in gynecologic oncology are highly specialized operations that are an important part of oncofertility practice. According to the recommendations of professional societies, they are considered safe in carefully selected cases [13]. The feasibility of these surgeries in early-stage ovarian, endometrial, and cervical cancer is not only enabled by timely diagnosis and better screening programs, but also by highly specialized gynecological surgery, advances in imaging techniques, better understanding of tumor biology, and availability of effective adjuvant therapy [14].

Despite the growing body of fertility-sparing surgery protocols and recommendations, many uncertainties, concerns, and challenges remain when balancing oncologic treatments and fertility preservation techniques. Delayed or absent referrals to reproductive specialists is a global issue, leading to missed reproductive opportunities and emotional distress [6,7].

Therefore, the primary study aim was to present our institutional practice (protocols and used fertility-sparing treatments) and describe our long-term experience (oncologic and obstetrical outcomes) concerning young female patients referred to the Clinic of Obstetrics and Gynecology University Clinical Centre of Serbia for fertility preservation.

Moreover, the secondary study aim was to assess the effectiveness of the oncofertility program at our institution, which offers both fertility-sparing surgical and medication treatment and cryopreservation.

## 2. Materials and Methods

This is a retrospective descriptive single-center cohort study that included all patients referred to the Clinic of Obstetrics and Gynecology (Ob/Gyn Clinic) University Clinical Center of Serbia (UCCS) for fertility-sparing treatment and preservation over ten years. Patients’ demographic characteristics, cancer type, treatment method, and oncologic and obstetrical outcomes were collected from medical records. The UCCS Ethics Committee (576/4, 622/3, 694/11) approved the study. The outcomes for conservatively treated patients with endometrial and ovarian malignancy have been thoroughly analyzed and reported in our previous studies, while data regarding cervical malignancy and cryopreservation of reproductive materials will be fully investigated in further research. Accordingly, in this study we presented data of malignancy-specific populations briefly and focused on evaluating the oncofertility program of our institution comprehensively.

### 2.1. Our Practice and Experience

At the UCCS and the Institute for Radiology and Oncology of Serbia (IORS), patients can receive comprehensive treatment in one place—from diagnosis and malignancy treatment to assisted reproduction, pregnancy management, and finally delivery. The Serbian national referral center for oncofertility and fertility preservation is based at the Ob/Gyn Clinic UCCS, where highly specialized fertility-sparing surgeries, cryopreservation, assisted reproduction (ART), perinatal care, and deliveries are performed. Radiotherapy and chemotherapy for malignancy as well as genetic testing are performed at IORS. For optimal malignancy treatment and long-term follow-up, collaboration is also realized with other corresponding specialists from UCCS.

Each female patient referred due to malignancy at a young age and who is still interested in reproduction is carefully evaluated and managed by a multidisciplinary team (Board for Cancer and Human Reproduction and Board for Gynecological Oncology at the Ob/Gyn Clinic UCCS), including gynecologists, medical oncologists, pathologists, and neonatologists. Decisions are also made in collaboration with other specialists according to malignancy type.

The criteria for fertility-sparing in our clinic correspond with the European Society of Gynecological Oncology, European Society of Human Reproduction and Embryology and European Society for Gynecological Endoscopy (ESGO/ESHRE/ESGE) and International Federation of Gynecology and Obstetrics (FIGO) guidelines and include malignancy with favorable oncologic criteria, age ≤ 45 years, a strong desire to preserve fertility, and signed informed consent. If patients have contraindications, or they decline fertility-sparing treatment, radical treatment is performed according to the current standard protocols [15,16].

The standard evaluation for patients preparing for fertility-sparing treatment includes thorough laboratory investigations (blood count, biochemical analyses, coagulation factors, and tumor markers), the Papanicolaou test (Pap smear), pelvic magnetic resonance imaging (MRI), chest X-ray, breast ultrasound, and, when indicated, genetic testing.

Ovarian function is evaluated using transvaginal gynecological ultrasound with antral follicle count (AFC), along with baseline hormonal status and anti-Müllerian hormone (AMH) that are determined before treatment. These assessments help understand a patient’s fertility prospects and available treatment options, as well as the future likelihood of requiring assisted reproductive techniques (ART) for pregnancy.

Fertility-sparing treatment in our institution is individualized according to clinical, histological, laboratory, and imaging findings, while strictly adhering to standardized FIGO and ESGO/ESHRE/ESGE guidelines [15,16].

Ovarian function is evaluated after therapy in patients who had tried to conceive. As no case of ovarian function failure was registered, all our patients were counseled to attempt pregnancy as soon as possible after complete disease remission was confirmed. In the case of ART the controlled ovarian stimulation (COS) protocol was determined on a case-by-case basis according to hormonal and ultrasound findings, cancer type, and the time available before treatment. According to guidelines, for hematological patients in whom chemotherapy had already begun, COS was performed six months after the last cycle [15,16].

The follow-up of conservatively treated oncofertility patients is regular and ongoing. They are followed up according to the current standard protocols based on malignancy type. For all malignancies, follow-up includes laboratory tests and imaging every three months during the first and second years, every six months until the end of the fifth year, and annually thereafter. For endometrial cancer, follow-up includes intrauterine device extraction and hysteroscopy every six months for uterine cavity evaluation and endometrial sampling. For cervical cancer, a Pap smear is performed every six months. More frequent check-ups were indicated for patients exhibiting symptoms or abnormal examination findings. In cases of recurrence, localization and time of recurrence, as well as subsequent treatment, were documented.

In this study, we analyzed the 10-year follow-up period. All presented patients were followed up for at least 12 months (average follow-up time in the overall sample 66.26 ± 28.34). At all control check-ups, their condition was assessed by clinical, laboratory and imaging methods and based on findings classified as good (complete remission, i.e., malignancy-free) or bad (disease persisting, progressing, or returning). Disease persisting represents no regression of the findings during the assessed period, regardless of therapy; disease progressing means stage or grade of the malignancy increasing during follow-up and disease returning represents malignancy diagnosed again after a period of complete remission.

Finally, all attempted and achieved pregnancies were recorded along with the time and method of conception. Patients regularly controlled their pregnancies in our institution, and we registered all complications (bleeding, contractions, gestational illnesses, growth restriction, fluid disturbances, etc.), adverse outcomes (miscarriage, intrauterine fetal death, stillbirth and early neonatal death), mode and gestational week of delivery.

The main outcome measures in this study were the patients’ oncologic and reproductive outcomes. The principal oncologic outcome was overall survival (the percentage of patients still alive after a specific period of time after diagnosis) and the secondary outcome was recurrence rate (the percentage of patients with disease recurrence after a period of remission).

The principal reproductive outcomes were pregnancy rates, while live-birth rates were secondary outcome measures. As rates for reproductive outcomes can significantly differ according to the used denominators, both pregnancy and live-birth rates were calculated and presented as overall (among all treated patients), and for patients who actually attempted conception.

### 2.2. Oncofertility Program Effectiveness

In addition, as the novelty of this study, we analyzed program-level indicators to measure the effectiveness of our oncofertility program (how well our oncofertility practice functions in everyday clinical settings and its broader real-world impact). These indicators included program input (staff-to-patient ratios, staff time, funding, and invested material resources); program output (the number of patients provided with counseling and the number of treatments delivered); treatment waiting time (the interval between diagnosis and treatment initiation); and program and condition-specific outcome (the percentage of oncofertility patients who adopted the recommended fertility-sparing treatment, adherence to clinical protocols, survival and mortality rates, and patient safety measures, such as complication rates and recurrence rates).

Clinically, indicators represent the measure of healthcare accessibility and quality, patient safety, and staff workload. Higher staffing levels directly correlate with lower rates of medical errors, hospital-acquired infections, patient complications, and mortality. High follow-up rates and low patient-lost-for-treatment rates show that patients receive continuous care essential for detecting early signs of treatment failure or disease progression and/or recurrence, managing medication side effects, and adjusting treatment before complications worsen. In contrast, prolonged treatment waiting times indicate a mismatch between patient demand and healthcare capacity causing lower patient satisfaction, poorer compliance with follow-up care, and potential progression of the disease. Finally, patient safety event rate shows how safely the care is delivered and can be a sign of adhering to protocols, communication, breaking work processes, and/or understaffing.

### 2.3. Statistical Analysis

The obtained data were analyzed using descriptive (number, percent, mean, and standard deviation) and analytical statistical methods (Kaplan–Meier survival analysis) in SPSS version 21. The program-level indicators were evaluated and measured using standard definitions and formulae which are presented in Table 1.

## 3. Results

**Figure 1:**
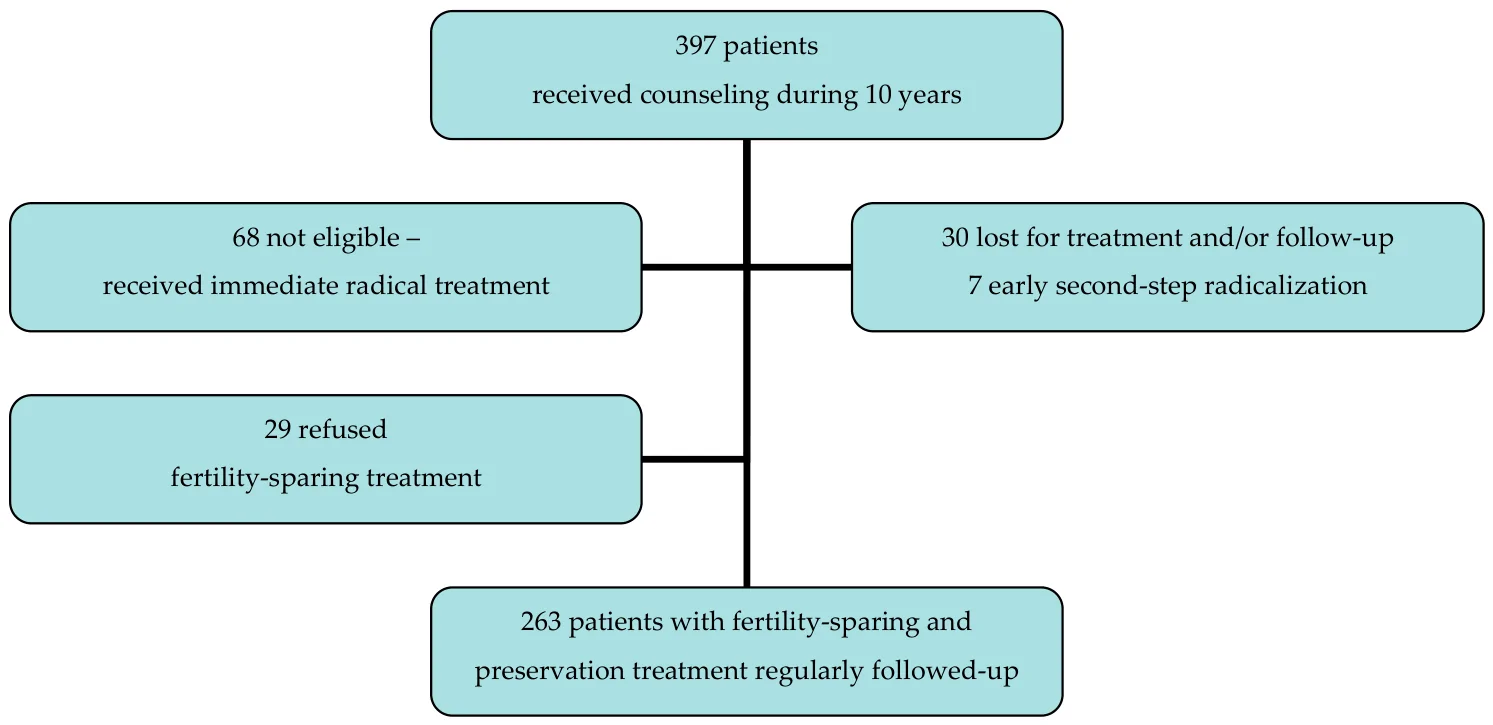
The study population.

There has been a general upward trend in the number of young patients diagnosed with malignant disease referred for fertility preservation over the years (Figure 1). The average annual number of counseled female oncofertility patients is around 40, out of which majority receives proposed fertility-sparing treatment. Consequently, in this study we analyzed data of 263 oncofertility patients conservatively treated during a ten-year period at the Ob/Gyn Clinic UCCS. Table 2 presents all fertility-sparing modalities that were analyzed in this study and Table 3 shows oncofertility program-level indicators. However, it should be pointed out that the study results should be interpreted only within the context of strict eligibility criteria and careful multidisciplinary selection of patients for fertility-sparing treatment.

Following current diagnostic and therapeutic protocols, we managed to obtain rather well both oncologic and obstetrical outcomes for our sample of oncofertility patients selected according to strict criteria (Table 4).

### 3.1. Cervical Cancer

Fifteen patients with early-stage cervical cancer were conservatively surgically treated by abdominal radical trachelectomy during a ten-year period in the Ob/Gyn Clinic UCCS, out of which the majority (73.3%) had squamocellular carcinoma and the remaining 26.7% had cervical adenocarcinoma. The average patients’ age was 31.2 ± 9.57 years and all were nuliparous. The majority of women had no comorbidities (75%), but 25% were overweight and obese.

All patients were evaluated preoperatively by MRI in order to meet the selection criteria for fertility-sparing treatment. Pathological diagnosis was obtained after biopsy in 46.7% and after cold-knife conisation or loop excision in 53.3% of patients. Definitive pathological analysis after trachelectomy confirmed carcinoma in 53.3% of patients, one had cervical intraepithelial neoplasm grade III, while no malignant cells were detected in the final cervical specimen of the remaining 40% of patients. Adjuvant chemotherapy was not administered in any of our patients.

All patients had bilateral extraction of pelvic lymph nodes which were subjected to ex-tempore frozen section histological examination. After confirmation of malignancy negative lymph nodes, cervical amputation was performed with at least 5 mm of the free endocervical margin. Parametrial resection was performed at least 10mm laterally of the cervix. Uterine arteries were conserved in all but two patients in whom they had to be resected. In all cases, surgical margins after trachelectomy were malignancy-free. Still, in one patient, a radical hysterectomy had to be performed due to a positive ex-tempore lymph node biopsy.

The mean follow-up time was mean 50.7 ± 31.7 months. Outcomes of our patients after follow-up were excellent as we registered no recurrent diseases and had just one adverse outcome (survival rate 93.3%). Trachelectomy had few complications. Cervical stenosis was observed in one patient and was associated with infertility and unsuccessful ART attempts. Another patient had lymphocele. During the first year of follow-up, one patient had metastases from the original cancer in the ovary. Another patient developed de novo ovarian cancer four years after cervical malignancy. She refused hysterectomy, so during the seventh year of follow-up, she had metastases of the ovarian cancer to the endometrium that were treated with adjuvant radiotherapy.

Only four women tried to conceive after treatment and they all succeeded. Moreover, one patient is currently pregnant for the second time. The overall pregnancy rate was 26.7% and in patients who actually attempted pregnancy it was 100%. They all underwent ART, but one pregnancy ended in an early miscarriage. The remaining three patients delivered healthy children by planned Cesarean Section in the 32–33 weeks of gestation. Consequently, the overall live-birth rate was 20%, while in patients who actually attempted pregnancy it was 75% (Table 5).

### 3.2. Endometrial Cancer

During a ten-year period, 87 patients with atypical endometrial hyperplasia (AH), intraepithelial endometrial neoplasm (EIN) and early-stage endometrial cancer were conservatively treated and followed up at the Ob/Gyn Clinic UCCS [17]. In addition, 12 new patients are currently on treatment while around 30 patients were lost for follow-up and therefore not presented. The average age of investigated patients was 35.05 ± 5.09 years, and they were nulliparous at 86.5%. The majority of women had no comorbidities (65.2%), but 44.8% were overweight and obese.

The initial diagnosis was made histologically after hysteroscopy and/or uterine curettage performed due to an endometrial polyp visualized by ultrasound, irregular bleeding or infertility. The most common histological finding was endometrial carcinoma, out of which 84.6% were of the first (G1) and 15.4% of the second (G2) grade.

Application of a levonorgestrel-releasing intrauterine device (LNG-IUD) was the treatment of choice for all investigated patients, along with gonadotropin-releasing hormone analogs, while additional therapy was administered in around a quarter of patients including lifestyle changes, metformin and hysteroscopic resection of endometrial lesions. These additional therapies were administered according to indications based on current guidelines and recommendations. The therapy was well tolerated with almost no adverse effects and, consequently, total compliance.

The mean follow-up time was mean 76.4 ± 30.6 months. Response to therapy was good with complete remission in 74.7% of patients and no adverse outcomes during the analyzed ten years of follow-up (survival rate 100%). Nevertheless, during the follow-up, recurrent disease was registered in 4.6% and progression from EIN to invasive cancer in 3.4% of patients. Finally, radical surgical treatment had to be performed in 14 patients (12 due to condition deterioration and 2 due to finishing reproduction).

After complete disease remission, 29.9% of patients attempted pregnancy, but only nine (overall 10.3% and in patients actually attempting pregnancy 34.6%) achieved clinical pregnancy (Table 5). Although the majority of patients underwent ART, 55.6% of pregnancies were conceived naturally. There were two early miscarriages, while the remaining seven pregnancies passed without major complications and ended in a term delivery of a healthy child, giving the overall live-birth rate of 8%, while in patients who actually attempted a pregnancy it was 26.9%. None of the patients who achieved pregnancy had progressive or recurrent diseases.

### 3.3. Ovarian Cancer

The decision to perform fertility sparing surgery (preservation of the uterus and at least a part of one ovary) in the case of adnexal masses was based on preoperative assessment that indicated borderline or low-stage disease and the absence of any other risk factors. Postoperatively, the final choice of therapeutic approach (follow-up, adjuvant chemotherapy or surgical radicalization) was made in accordance with histological findings.

During a ten-year period, 39 patients with non-epithelial ovarian carcinomas (NEOC) were conservatively treated at the Ob/Gyn Clinic UCCS [18]. They, on average, had 26.87 ± 6.95 years of age, and were mostly were nulliparous (66.7%). Majority of women had no comorbidities (67.8%), but 43.2% were overweight and obese.

The most frequent histological findings were granulose-cell tumor (53.8%) and dysgerminoma (20.5%) while immature teratoma, yolk sack and Sertoli Leidig tumors were less frequent.

Unilateral salpingo-oophorectomy was therapy of choice for 30.8% of patients. Cytology testing and omental biopsy was performed for all staged patients, biopsy of the contra-lateral ovary and peritoneum for about 40% of staged patients, while lymphadenectomy was performed for only six patients. The majority of patients had one-step staging, but 28.2% of patients also had second-step surgery. Adjuvant chemotherapy received 48.7% of patients (two up to six cycles of Bleomycin–Etoposide–Cisplatin regimen).

The mean follow-up time was 62.92 ± 45.63 months. The overall survival rate at the end of ten-year follow-up was 87.2% (five adverse outcomes occurred in 9 to 38 months). Recurrence of NEOCs was registered in a quarter of patients, mostly during the first two postoperative years, with the highest recurrence rate for Sertoli Leydig and granulosa cell tumors. This outcome might have been influenced by the higher stage (IC in 50%) of these two malignancies at the time of diagnosis. Surgical radicalization due to recurrence was performed in four patients while nine patients received additional chemotherapy.

Following initial treatment, 25.6% of patients tried to conceive but only 7 (18%) succeeded (Table 5). Only one NEOCs patient achieved pregnancy by ART, while others conceived spontaneously. No miscarriages were registered and pregnancies were uneventful. Two children were born preterm, but all were live-born singletons in good condition upon birth (overall live-birth rate 18% and in patients who actually attempted pregnancy 70%).

Three patients were treated with chemotherapy prior to pregnancy achievement (two patients two years and one four years after treatment). Out of patients with recurrent disease, only one with dysgerminoma spontaneously achieved pregnancy before recurrence.

During a ten-year period, 52 patients with stage I borderline ovarian tumors (BOT) were treated at the Ob/Gyn Clinic UCCS with the mean age of 32.6 ± 5.9 years [19]. They were mostly nulliparous (63.5%). The majority of women had no comorbidities (85%), but 46.0% were overweight and obese.

The most common histological type was serous BOT (52%) patients, followed by mucinous histology (42%) and endometrioid BOT in three patients. More than 2/3 of tumors were in stages IA and IC1.

Unilateral salpingo-oophorectomy (57.8%) was the most common primary surgery. The surgical staging was performed as a first-step procedure in more than half of the patients (55.8%) including peritoneal wash cytological examination (100%) assessment of the omentum (94.2%), contra-lateral ovary (59.6%), lymph nodes and appendix (in 10 and 3 cases, respectively).

Upon staging, in six patients, the initial cystectomy was followed by the removal of the involved ovary during the second-step procedure. In addition, radical surgery had to be performed in 7 patients as the second intervention, leaving 45 patients on fertility-sparing treatment and follow-up. In all patients who had second-step surgery, no residual atypical or malignant ovarian tissue was found. Adjuvant chemotherapy was administered in 21.2% of patients (up to six cycles of Carboplatin monotherapy and Carboplatin/Taxol in one patient).

The mean follow-up time was 73.4 ± 35.29 months. All patients were alive at the end of ten-year follow-up, giving an overall survival rate of 100%. However, during the follow-up, recurrences were registered in 7.7% of patients, mostly during the first three postoperative years.

During the follow-up, 64.4% of patients tried to conceive and 44.8% succeeded (all naturally except one case of ART). Pregnancies occurred one to seven years after BOT diagnosis. No miscarriages were registered and pregnancies were uneventful. All children were live-born, term singletons and in good condition at birth (overall live-birth rate 25% and in patients who actually attempted pregnancy 44.8%).

In the Ob/Gyn Clinic UCCS ovarian transposition was performed in three patients prior to radiotherapy due to cervical cancer as a tentative fertility-sparing method for potential preservation of the ovarian function and a source of oocyte aspiration for use in attempting a surrogate pregnancy. However, none of these patients attempted further pregnancy as currently surrogate pregnancy is not legal in Serbia (Table 5).

### 3.4. Cryopreservation

During a ten-year period, 80 female patients were referred to cryopreservation of reproductive materials at the Ob/Gyn Clinic UCCS (Table 6). However, the procedure was discontinued in two patients due to deterioration of the malignant disease. One more patient discontinued the procedure for personal reasons. Consequently, data regarding 77 female oncofertility patients were presented. Patients in average had 32.93 ± 5.98 years of age. They were mostly nulliparous (88.9%) and without comorbidities (61.7%). The majority of women had no comorbidities (67.5%). In our sample, 28.6% of patients were overweight, but no patients were obese.

The most common malignancy due to which reproductive materials were cryopreserved was breast cancer (n = 38), followed by gynecological and hematological malignancies. Hematological malignancies were more common among women younger than the age of 30 years, while breast cancers were generally found in patients aged 31 to 40 years.

COS was initiated conventionally at the beginning of the cycle for the majority of patients (57.1%). Gonadotropins were administered for ovarian stimulation in the majority of oncofertility patients (73% as a single agent and with aromatase inhibitors in an additional 3% of cases) on an average of 9.66 ± 4.99 days. Up to 28 oocytes per patient were obtained (average 9.19 ± 8.33) out of which the majority were successfully cryopreserved (average 8.22 ± 8.37) (Table 7).

In our institution, oocytes were generally cryopreserved while embryos were preserved in six cases. Moreover, there were eight cases of ovarian tissue cryopreservation that were performed during surgical treatment of gynecological and breast cancers. The average duration of storage for presented patients was 4.37 ± 1.2 years, but all of our patients still have cryopreserved reproductive materials in our institution.

After successful malignancy treatment, three patients attempted pregnancy by ART (one with embryos and two with oocytes) giving a cryopreserved material utilization rate of 4.5%. Out of them, two clinical pregnancies were achieved. One pregnancy successfully ended in the delivery of a healthy child, while the other ended in an early miscarriage. Consequently, the overall pregnancy rate was 3%, while in patients who actually used the cryopreserved material it was 66.7%. The overall live-birth rate was 1.5% and in patients who actually attempted pregnancy it was 33.3%.

## 4. Discussion

This study summarizes clinical experience of a national referral oncofertility center where diverse multidisciplinary fertility-sparing treatments and cryopreservation of reproductive materials are performed within the same institutional framework. It is novel in assessing the effectiveness of the oncofertility program at the Ob/Gyn Clinic UCCS for all young patients diagnosed with malignant conditions. It should be emphasized that obtained results are for the overall group of patients with cervical, endometrial and ovarian malignancies. This heterogeneity of the study population should be taken into consideration when interpreting measures of program success. The presented aggregate program-level indicators prevent direct comparability among the different disease groups but rather show the multidisciplinary scope of our institution.

### 4.1. Cervical Cancer

Although the incidence of cervical cancer is decreasing in developed countries, around 20% is still diagnosed in women who have not completed reproduction [20]. For early-stage cervical cancer of such patients, the preferred treatment is trachelectomy as a fertility-sparing option. The selection criteria for trachelectomy are based on clinical examination and imaging techniques as well as histological type (all grades of squamous cell carcinoma and usual-type HPV-associated adenocarcinoma), which are confirmed by cervical biopsy or conisation [21]. Recent evidence highlights the importance of immunohistochemical assessment for patients with cervical squamous cell carcinoma for better patient selection and outcomes prediction [22]. According to current guidelines, simple trachelectomy is possible in patients with FIGO stage IA1 or IA2, regardless of limphovascular invasion (LVSI), as well as in those with stage IB1 and LVSI-negative tumors. Radical trachelectomy is performed in patients with stage IB1 and LVSI-positive tumors [23]. For patients with stage IB2 (tumor 2–4 cm), which carries a high recurrence risk, fertility-sparing surgery remains controversial. Finally, free margins on the final pathology after trachelectomy must be obtained [21]. It can be seen that there is significant heterogeneity in management and no standard treatment. Thus, fertility-sparing treatment may be considered in selected patients after an in-depth discussion with the patient and the gynecologic oncology team [24,25].

The recurrence rate after abdominal radical trachelectomy is 3.3% to 4% (range 0–25%), which, according to recent studies, is lower than after vaginal radical trachelectomy. The time to recurrence is mostly similar for abdominal and vaginal surgical approaches (around 2 years), while recurrence occurs sooner with the laparoscopic approach. The overall five-year survival rate in previous studies was generally high and similar for abdominal and vaginal trachelectomy (95–99%) [26]. In our sample, no patients had recurrent disease.

According to the literature, pregnancy rates after trachelectomy are lower than those in the general population. In a large study including 230 patients, the pregnancy rate was 41%, while in one case series including 36 patients, the rate was 44.5% [27,28]. Another investigation reported that 62% of patients who underwent trachelectomy tried to conceive, out of which 73% succeeded and had a high live-birth rate [29]. Moreover, uterine artery preservation has been proven to be the surgical parameter associated with a higher live-birth rate [20]. We generally had a small sample of patients with trachelectomy, out of which only a few tried to achieve pregnancy. However, the pregnancy rate among them was 100% and the live-birth rate was 75%.

### 4.2. Endometrial Cancer

Fertility-sparing hormonal treatment for AH, EIN, and early-stage endometrial cancer has been proven to be both oncologically effective and safe. Although the analyzed histological subtypes differ, the treatment and follow-up are the same, enabling assessment as a single category. Qualified patients are younger than 40 years with a premalignant disease or a well-differentiated endometrioid cancer confined to the endometrium and who are highly motivated to preserve their reproductive function. Their evaluation is based on the histological findings of the endometrial samples, along with MRI and/or transvaginal ultrasound images, to assess myometrium invasion and extrauterine spread. Additional genetic testing is suggested [30,31].

Currently, endometrial cancers are divided into subtypes according to immunohistochemistry and molecular techniques. The subtype with mutations in the polymerase epsilon gene (POLE) has the best prognosis, the DNA mismatch repair-deficient (dMMR) subtype has an intermediate prognosis, while the subtype with abnormal p53 protein has the worst prognosis. This classification is also important for appropriate adjuvant therapy. Patients with abnormal p53 protein should have intensive adjuvant therapy, while those with mismatch deficiency advanced or recurrent cancer should receive immunotherapy [15,32]. We have recently started to perform genetic testing and consider molecular classification when choosing the treatment for endometrial cancer patients.

Oral progestogens (medroxyprogesterone) are recommended as the first-line treatment to antagonize estrogen [33,34]. GnRHa could be an alternative therapy, as it exhibits protective effects on ovarian reserve, contributing to improved pregnancy rates [35]. Using LNG-IUD is suggested to deliver a high concentration of progesterone directly to the endometrium while acting only locally and thereby evading side effects [30]. Consequently, the combination of LNG-IUD with oral progestogens and/or GnRHa is currently the treatment of choice, as it significantly enhances treatment effectiveness [30,31,32,33,34]. Our patients received LNG-IUD combined with GnRHa, which was proven to be safe, effective, and comfortable. Additional therapies (metformin, lifestyle changes) were administered according to the relevant guidelines only to patients who met the indications for such therapy.

The rates of complete remission vary between studies and institutions, but they are generally high (mostly above 50% and even reaching 100%). Optimal remission (96.3%) is achieved by using combined therapeutic modalities (hysteroscopic resection of visible tumor followed by hormonal therapy) [36,37]. However, recurrence rates are mostly high and should not be underestimated (7.7–89%) [38,39]. In the case of complete remission, long-term survival is relatively good, but if recurrence or progression is diagnosed, the outcomes are generally adverse [30,39]. We achieved complete remission in approximately 75% of patients.

The literature shows that conception and live-birth rates are promising. The reported rates range from 20% (when all treated patients are included) to 93% (when only those who attempted conception are considered). However, generally, only around 50% of patients with complete endometrial cancer remission attempt to conceive, although pregnancy can positively impact the prognosis of endometrial cancer [39,40,41,42]. In our sample, only around 30% of patients attempted pregnancy, but of them 34.6% achieved pregnancy.

### 4.3. Ovarian Cancer

Fertility-sparing surgery is generally reserved for patients with early-stage ovarian malignancy, i.e., a well-encapsulated cancer confined to one ovary, with no uterine involvement, no spread to the pelvis or abdomen, and negative peritoneal washings. In practice, fertility-sparing surgery is mostly performed for borderline ovarian tumors, malignant germ cell tumors, sex cord-stromal tumors (SCST), and strictly selected patients with early-stage, low-grade epithelial ovarian cancer [43,44,45,46].

Depending on the tumor’s size, stage, and type, fertility-sparing surgery typically involves ovarian cystectomy (for borderline or benign-appearing tumors) or unilateral adnexectomy. Accurate MRI-based preoperative assessment of adnexal masses is essential to ensure that fertility-sparing surgery is performed appropriately [43,44,45,46].

The literature indicates that five-year overall survival after fertility-sparing surgery is high if patients are adequately selected for such an approach (above 80%). Survival rates are similar regardless of tumor type, except for SCST, which has a worse prognosis. However, survival was found to be similar between patients treated with fertility-sparing and radical surgery even for SCST [47,48,49]. In our sample, survival rate was almost 90%.

Compared to radical treatment, fertility-sparing treatment is associated with a higher recurrence rate, but most recurrences can be managed by further surgery. In the case of recurrence, the prognosis is good if the recurrence is limited to the preserved ovary, while it is poor if it occurs outside the preserved ovary, though this is less common [49,50,51,52,53].

The observed pregnancy rates after fertility-sparing treatment for patients with borderline ovarian malignancies vary between 32% and 88% [47,48,54,55]. As for early-stage ovarian carcinoma, the data are scarce, but they indicate that out of the 42% of patients who tried to conceive after fertility-sparing surgery, 67% achieved pregnancy, resulting in high live-birth rates [56,57]. Previous studies regarding NEOC patients revealed both high pregnancy (86.4%) and live-birth rates (95%) [49,50,58]. This was also true for our sample (all patients who were conservatively treated for ovarian tumors and achieved pregnancy delivered a healthy child).

### 4.4. Cryopreservation

Fertility preservation in oncologic patients is not limited to fertility-sparing surgery. Cryopreservation of reproductive material represents the second major component of the oncofertility program [59].

Although there have been concerns about estrogen exposure during ovarian stimulation and its effect on malignant disease, the literature confirms the safety of this approach, even in hormone-sensitive cancers [60]. The process of COS for cryopreservation itself, as well as the use of gonadotropins for COS, does not significantly impact both the six-month and long-term survival rates of patients regardless of malignancy type. The use of aromatase inhibitors combined with gonadotropins reduces estrogen levels and represents an extremely safe stimulation method for hormone-dependent tumors [61,62]. The use of GnRH agonists as a trigger is particularly recommended due to the lower risk of ovarian hyperstimulation, which should be strictly avoided [60].

Some studies have suggested that oncology patients undergoing COS for fertility preservation respond well (65% of patients had six or more oocytes), similar to other infertility patients with the same characteristics who underwent the same COS protocol [63,64]. In contrast, a few large studies and meta-analyses reported that oncofertility patients produced fewer oocytes compared to healthy age-matched controls, while other studies did not find any significant differences in the number of retrieved oocytes [65,66]. Although the overall ovarian response to COS in our sample was satisfactory, a relatively high percentage of oncofertility patients were classified as poor responders to stimulation. This may indicate the existence of additional factors (genetic predispositions or subclinical impairment of ovarian function) that demand further analysis [63,64].

Ovarian stimulation and oocyte cryopreservation, however, are not feasible for prepubertal girls. For these patients, ovarian tissue preservation is ideal, although the risk of reintroducing malignant cells after ovarian tissue transplantation remains under debate, especially in patients with leukemia [62,67]. In some cases, a patient’s general medical condition can also be a concern for COS. Oncofertility patients are sometimes critically ill, with reduced cardiopulmonary, renal, and hepatic function, and complications such as infection, bleeding, and thrombosis. Moreover, patients with cytopenia and thrombocytopenia are not eligible for fertility-sparing procedures [62,67].

### 4.5. Oncofertility Program Effectiveness and General Remarks

The global average mortality rate for all cancers in young female patients (ages 15–39) is 13.2 per 100,000. The average mortality rate for hospitalized oncologic patients is around 5%, but institutions should aim for a lower percentage (optimally under 2%). Leading causes are breast cancer, cervical cancer, and leukemia [68]. The mortality rate of young patients with gynecological malignancies that we treated and followed was 2.3%.

It is important to highlight that fertility-sparing treatment is still not considered standard care. It is only a temporary measure with the main goal of delaying definitive radical surgery to enable childbearing. Consequently, therapy should start immediately after diagnosis, while patients should be encouraged to conceive as early as possible after disease remission. Definitive surgery is strongly indicated after childbearing to prevent tumor recurrence [13,14,69].

One issue that should be considered when assessing the success of an oncofertility program are generally low pregnancy-attempt rates among remission patients and very low post-thaw utilization of cryopreserved material. Usage rates of cryopreserved materials for cancer patients in the literature range from 3.1% to 30%. This was true for our patients as well, where less than 50 percent of patients attempted pregnancy after treatment. However, such findings do not necessarily imply a low effectiveness of cryopreservation. These rates vary between different populations, and depend on the duration of the follow-up period and the time of the assessment, as many patients need prolonged periods of time to complete cancer treatment. The most common reasons include general health and disease-related parameters, patient mortality, current desire for pregnancy, spontaneous natural pregnancies, financial constraints, or changes in socio-demographic circumstances (relationship status) [70]. Therefore, standard follow-up care for cancer patients typically lasts for at least five years. The follow-up of our patients is ongoing and here we present the data for the first ten years of follow-up. During this period, we had 10.2% that were lost for follow-up. The optimal loss to follow-up rate in clinical research is generally considered to be fewer than 5%, while a rate above 20% can impair the validity of conclusions [71]. Consequently, we had a moderate loss of patients, which could be explained by the prolonged follow-up. There were no differences regarding malignancy or socio-demographics between patients regularly followed up or lost for follow-up. Majority were just lost due to long follow-up period.

To improve cancer survival, women’s health, and quality of life, a comprehensive approach through national fertility institutions must be established as a national priority, supported by international associations that offer constant clinical practice guidelines [8,9]. Generally, the recommended staff-to-patient-ratio for the surgical department is 4:1 or 5:1. In academic medical intensive care units, an intensivist-to-patient ratio less than 1:14 may negatively impact education, staff well-being, and patient care [72]. Although there are currently no available data for oncofertility departments, such facilities should have a staff-to-patient ratio similar to surgical facilities. In our oncofertility department, the ratio of doctors and patients treated annually corresponds with the recommended (4.6:1).

Additionally, the literature shows that the lack of a systematic referral pathway is another reason for low fertility preservation uptake [73,74,75,76]. Healthcare professionals worldwide are generally not fully aware of the importance of a multidisciplinary approach to oncofertility. Although 79% of doctors often or always discuss fertility preservation options with their patients, only 50% of patients are referred to reproductive specialists [72]. Counseling should occur as soon as possible to ensure that fertility preservation can proceed immediately without delaying cancer therapy [2,69]. It is considered that for an adequately organized cancer care, the treatment should start within 30-60 days from diagnosis. In the systematic review and meta-analysis, pooled mean delay from diagnosis to treatment for cervical carcinoma patients worldwide was 71.42 days [76]. In a study regarding patients with AH, only 18% had the recommended 62-day referral to treatment time while 56% managed to be treated within the recommended 31 days after diagnosis [77]. In our institution the treatment waiting time was mostly within one month, but for all of our patients it did not exceed two months.

According to guidelines, an oncofertility program requires a multidisciplinary approach and should aim to overcome barriers regarding access and care for different types of patients, while complying with country legislation. Consequently, oncofertility programs differ between centers and countries because of variations in the organization of clinical care and legal frameworks [74,78]. Of 30 European countries, half offered cryopreservation of reproductive materials at no cost to patients, although in some of these countries specific conditions must be met to qualify for full reimbursement [78]. In some other countries, treatment costs are partially covered and only for married women under 40 years. However, this coverage is insufficient, especially for women who do not meet the eligibility criteria. The high costs of fertility-sparing treatment can deter many women from pursuing fertility preservation [73,78].

Serbia has a publicly funded universally available healthcare system which also provides the framework for cancer care. The healthcare system is organized into three formal tiers: primary healthcare centers that serve as the first point of contact (general practitioners and some specialist services including gynecology), secondary healthcare (general hospital and specialist services) and tertiary healthcare (University-affiliated Clinical Centers and specialized institutes for complex diagnostic and therapeutic procedures) [79].

Oncofertility services in Serbia are almost entirely concentrated in tertiary academic centers. A newly diagnosed cancer patient, regardless of age and reproductive history, is generally identified and evaluated at the primary or secondary level, but must then be referred onward to specialized tertiary centers for both oncologic treatment and further fertility preservation assessment [80].

In Serbia, oncofertility care began a decade ago and is currently organized and centralized in several specialized regional oncofertility institutions. In 2022, the Ob/Gyn Clinic UCCS was appointed by the Ministry of Health as the national referral center. The oncofertility team of the Ob/Gyn Clinic UCCS aims to ensure adequate and timely referral of all oncofertility patients from across the country to specialized centers by regularly educating Ob/Gyn specialists through seminars and lectures. Moreover, all patients are thoroughly informed about their condition and counseled about all therapeutic options (classic radical and fertility-sparing) [80].

In Serbia, fertility-sparing treatment and preservation are covered by state healthcare for women diagnosed with malignant diseases who are younger than 40 years, have preserved ovarian function, and have an early-stage malignancy without metastases at the time of diagnosis. The approach to patients is individualized according to their age, parity, comorbidities, malignancy histology, stage, and current relationship status. Both diagnosis and surgical therapy can be repeated as many times as necessary at no cost to patients, whereas oncofertility patients are entitled to one COS procedure with cryopreservation of reproductive material before oncologic treatment begins. Finally, according to Serbian legislation, storage of cryopreserved reproductive material of oncofertility patients is indefinite [80].

To our knowledge, this is the first study to assess the effectiveness (uptake, protocols, and outcomes) of oncofertility programs in Serbia. It showed positive results in terms of the organization of the oncofertility program and patient outcomes within this carefully selected population. Our findings regarding organization and protocol adherence can be transferable to other oncofertility centers, but issues with funding in different settings should be taken into consideration when interpreting and interpolating the study results.

This study has several limitations, including selection and referral bias that might have influenced excellent oncologic and reproductive outcomes. We collected data only from a single highly specialized oncofertility referral center. Similar multicenter experiences should be presented in future studies. Moreover, the cohort consists of highly selected young and low-risk malignancy patients who have not yet finished their reproduction. However, according to current guidelines, only such patients are candidates for fertility-sparing treatment in our setting. Therefore, the study findings are not generalizable to the broader population, but only to oncofertility patients with strict indications for fertility-sparing treatment. Moreover, legislation regarding oncofertility in Serbia is different from other countries and some of them (surrogacy prohibition in Serbia) might cause constraints for our patients, which can also impact the study results in terms of reproductive outcomes.

Furthermore, the study population is highly heterogeneous, comprising patients with malignancies that differ markedly in biological behavior, treatment strategies, and prognosis. Deriving general conclusions is therefore difficult. Evaluating the outcomes of different malignancies and fertility-preserving procedures together may influence the recurrence and reproductive outcomes. However, presenting fertility-sparing options for young patients with malignancies of different origins and histology was the very aim of our study: We assessed the effectiveness of the oncofertility program as a whole instead of condition-specific outcome predictors. We have already presented in our previous manuscripts the outcomes of treatment per malignancy type and its subgroups, along with the factors that impact patient outcomes. Finally, the histological type of endometrial or ovarian malignancy was not correlated with either disease recurrence or pregnancy achievement [17,18,19]. Moreover, the other studies that evaluated the effectiveness of therapeutic programs also assessed the similarly treated conditions together.

No inferential comparisons were intended in this research because of the heterogeneous populations and descriptive study design. Nevertheless, for further in-depth patient data analysis, we did apply multivariable regression to assess factors that impact oncological and reproductive outcomes. However, these results were thoroughly presented in our previous studies. Therefore, to avoid overlapping, we opted to present in this manuscript only the findings related to the current study aim.

Another limitation is the relatively small number of patients, especially in some malignancy subgroups. Few events upon which conclusions are derived can present a bias for results interpretation, as the calculated rates can be uncertain and over-interpreted. Since patients were investigated for a decade, some were expectedly lost in the follow-ups. Additionally, having a malignancy at a young age is fortunately a rare event, so it is difficult to obtain a large sample from a single institution. Our sample is therefore comparable in size to samples from other single-center studies [28].

Long-term follow-up is essential to collect detailed data, comprehensively evaluate patient outcomes, and report all recurrences, including late ones [10,37]. Consequently, the follow-up of our patients is ongoing and has extended to a decade for some. However, in our previous investigations, we found that short-term outcomes (recurrence and progression) are the most important, as short disease-free survival and early recurrence could compromise future fertility. Moreover, women who had completed childbearing underwent radical treatment, which consequently led to the fact that their outcomes changed in the sense that further prognosis was only associated with radical treatment.

Finally, we have to point out that some data regarding our patients have already been published which might pose a risk of duplicate publications. However, to avoid data overlapping, we now present only the findings relevant to the study aim. In addition, the approach in this investigation differs from previously published studies. It is more comprehensive as it includes and assesses all gynecological malignancies together in young patients treated by fertility-sparing procedures. Moreover, in the current study we evaluated not only patient outcomes but also the success of the implemented oncofertility program.

## 5. Conclusions

This study reports experience with the conservative treatment of oncofertility patients from a single national oncofertility referral center. The successful oncologic and obstetrical results support the feasibility of achieving favorable oncologic and reproductive outcomes in appropriately selected patients by following current treatment protocols. The approach to oncofertility patients should be integrated and multidisciplinary, but only for carefully selected low-risk malignancies in young patients desiring future pregnancy. However, our findings should be cautiously interpreted, as the study reflects the results of a specialized oncofertility referral center with rigorous patient selection based on strict, guidelines-based indications.

## Figures and Tables

**Table 1:** Assessed program-level indicators definitions and calculations.

Definition	Calculation
staff-to-patient ratio—the number of healthcare workers available to care for a specific number of patients	number of staff members/number of patients on unit during the assessed time period
average treatment waiting time—the mean duration a patient spends from an initial referral or clinic arrival to receiving the medical treatment	Σ (date of treatment − date of arrival per patient)/number of patients
counseling time (total and staff time)—total duration of direct therapeutic care a patient receives versus the working time spent by healthcare staff	total: Σ (number of sessions × duration of sessions) staff: total/number of staff members
patient follow-up rate—the percentage of eligible patients who successfully completed scheduled treatments and/or check-ups within a required timeframe	(number of patients who completed follow-up/total number of patients scheduled for follow-up) × 100
patients lost for treatment rate—rate of patients who failed to return for treatment or check-ups	(number of patients lost for follow-up/total number of patients who started the treatment) × 100
patient mortality rate—measures the frequency of death in a specific patient group over a given time frame	(total number of patient deaths/total number of patients with a condition) × 100
patient safety event rate—the frequency at which medical errors, adverse events, or near misses occur relative to a specific population, time frame, or unit of exposure	(total number of safety events reported during assessed period/total number of patient days over the same period) × 1000
invested material resources—equipment acquired and utilized to establish, execute, or sustain a healthcare program	

**Table 2:** Fertility-sparing procedures at Ob/Gyn Clinic UCCS and IORS.

Fertility-sparing surgery	Cervical cancer—trachelectomy—15
	Endometrial cancer—hysteroscopic resection—7
	Ovarian cancer—cystectomy/oophorectomy/adnexectomy—91
	Pelvic malignancy—ovarian transposition—3
Hormonal therapy	Endometrial cancer—LNG-IUD and GnRHa—87
Cryopreservation program	Oocyte cryopreservation—63
	Embryo cryopreservation—6
	Ovarian tissue—8

**Table 3:** Oncofertility program-level indicators.

Oncofertility department staff	12 (7 Ob/Gyn specialists and 5 residents)
Additional collaborators from other institutions	22 (specialists of oncology, internal medicine, etc.)
Patients provided with counseling (presented 10 years)	397
New patients provided with counseling (annual average)	40
Patients refusing suggested treatment (presented 10 years)	29
Patients adopting suggested fertility-sparing and preservation treatment (presented 10 years)	368
Initial radical treatments (presented 10 years)	68
Delivered fertility-sparing and preservation treatments (presented 10 years)	300
Delivered fertility-sparing and preservation treatments (annual average)	30
Patients lost for treatment and/or follow-up	30
Early second-step radicalizations	7
Average oncologic treatment waiting time	27.6 days (3 ± 1 week)
Oncofertility department staff-to-10-years-patient ratio	12/263 = 4.6, i.e., 1:22
All collaborators staff-to-10-years-patient ratio	34/263 = 12.9, i.e., 1:7.7
Oncofertility department staff-to-patient ratio annually	12/55 (40 new cases and 15 follow-ups) = 0.2, i.e., 1:4.6
All collaborators staff-to-patient ratio annually	34/55 = 0.6, i.e., 1:1.6
Counseling time (overall and staff time)	25 h weekly (2.1h per doctor)
Patient Safety Event Rate, i.e., complications rate	62 complications:5180 hospital days × 1000 = 12
Patient Follow-Up Rate	89.8%
Overall patient mortality rate all malignancies	2.3%
Funding	covered by state healthcare with no additional funding
Invested material resources	standard medical equipment and supplies, general laboratory, histological laboratory, artificial reproduction laboratory, genetic laboratory, cryopreservation equipment, imaging machines (US, X-ray, CT and MRI), medications, chemotherapy, radiotherapy

**Table 4:** Outcomes for fertility-sparing surgery, hormonal therapy, and cryopreservation program.

Fertility-Sparing Method/Outcome	Oncologic Outcome, i.e., Survival Rate	Recurrence Rate	Attempted Pregnancy Rate	Achieved Pregnancy Rate
Surgery for cervical cancer	93.3%	0%	26.7%	100%
Surgery for ovarian cancer	87.2%	19.8%	46.4%	51.3%
Hormonal therapy for endometrial cancer	100%	4.6%	29.9%	34.6%
Cryopreservation program	98.5%	0%	4.5%	66.7%

**Table 5:** Frequency of patients treated by fertility-sparing methods and their outcomes.

Parameters/Patient Number		Cervical Cancer *n* = 15	Endometrial Cancer *n* = 87	Ovarian Cancer *n* = 91
Histopathological diagnosis	squamocellular	11	/	/
	adenocarcinoma	4	/	/
	atypical hyperplasia	/	24	/
	EIN	/	24	/
	endometrioid	/	39	/
	NEOC	/	/	39
	borderline	/	/	52
Initial therapy	surgery	15	7	91
	hormonal	/	49	/
	chemotherapy	/	/	20
	radiotherapy	/	/	/
	intrauterine device	/	87	/
	other	/	12	/
Initial therapy response	positive	14	65	84
	negative	1	22	7
Follow-up	uneventful	11	74	46
	complications	4	13	45
Recurrence	no	15	83	77
	yes	0	4	14
Disease progression	no	13	76	86
	yes	2	11	5
Final patient condition	stable or healthy	14	65	69
	still ill or diseased	1	22	22
Pregnancy attempted	no	11	61	45
	yes	4	26	39
Pregnancy achieved	no	0	17	19
	yes	4	9	20
Pregnancy outcome	miscarriage	1	2	0
	live-born child	3	7	20

Legend: EIN—endometrial intraepithelial lesion; NEOC—non-epithelial ovarian carcinoma.

**Table 6:** Cryopreservation of reproductive material in Ob/Gyn Clinic UCCS in past 10 years.

Material	Malignancy Type	Patients Number	Pregnancy Attempted	Pregnancy Achieved	Live Birth
Embryo	cervical	1	/	/	/
	ovarian	1	1	1	1
	breast	3	/	/	/
	thyroid	1	/	/	/
Oocyte	gynecological	12	2	1	/
	breast	34	/	/	/
	hematological	13	/	/	/
	other	4	/	/	/
Ovarian tissue	cervical	4	/	/	/
	ovarian	3	/	/	/
	breast	1	/	/	/

**Table 7:** Number of retrieved and cryopreserved oocytes.

Oocytes per Patient		Retrieved		Cryopreserved	
		Mean	Standard Deviation	Mean	Standard Deviation
Malignancy type	gynecological	10.58	9.15	10.08	9.11
	breast cancer	7.85	7.81	6.67	7.88
	hematological	9.38	7.85	8.46	7.65
	other	15.75	11.29	15.01	11.16
Stimulation start time	spontaneous	2.33	3.83	0.51	0.84
	late follicular	6.33	6.81	4.01	3.61
	luteal phase	7.67	7.77	7.33	7.53
	classic	11.33	8.63	10.31	8.89
Stimulation protocol	spontaneous	2.33	3.83	0.51	0.84
	antagonist	9.59	8.08	8.69	8.07
	long	28.01	0.01	28.01	0.01
Stimulation medications	none	2.33	3.83	0.51	0.84
	gonadotropins	10.65	8.65	10.01	8.69
	aromatase inhibitors	5.01	5.53	3.12	4.79
	both	11.67	7.51	10.01	5.57

## Data Availability

The original contributions presented in this study are included in the article. Further inquiries can be directed to the corresponding author.

## References

[1] James E., Jenkins T.G. (2018). Epigenetics, infertility, and cancer: Future directions. Fertil. Steril..

[2] Benor A., Lindner P., DeCherney A.H. (2023). Editorial on infertility and cancer incidence. Fertil. Steril..

[3] International Agency for Research on Cancer (IARC) Cancer Today. https://gco.iarc.fr/today/en/dataviz/pie.

[4] Oncofertility Consortium. https://oncofertility.msu.edu.

[5] Park S.J., Han J.Y., Kim S.W., Kim H., Ku S.Y. (2024). Current position of oncofertility in adolescent female cancer patients: A comparative review on society guidelines. In Vivo.

[6] Jeruss J.S., Woodruff T.K. (2009). Preservation of fertility in patients with cancer. N. Engl. J. Med..

[7] Su H.I., Lacchetti C., Letourneau J., Partridge A.H., Qamar R., Quinn G.P., Reinecke J., Smith J.F., Tesch M., Wallace W.H. (2025). Fertility preservation in people with cancer: ASCO guideline update. J. Clin. Oncol..

[8] Robson D., Phua C., Howard R., Marren A. (2020). Fertility preservation in oncology patients: A literature review examining current fertility preservation techniques and access to oncofertility services in Australia. Aust. N. Z. J. Obstet. Gynaecol..

[9] Anderson R.A., Amant F., Braat D., D’Angelo A., Chuva de Sousa Lopes S.M., Demeestere I., Dwek S., Frith L., Lambertini M., Maslin C. (2020). ESHRE guideline: Female fertility preservation. Hum. Reprod. Open.

[10] Barioni J.C., Gozzo T.O. (2024). Fertility preservation counseling for women of reproductive age diagnosed with cancer: An integrative review. JBRA Assist. Reprod..

[11] Han J., Park S., Kim S.H. (2022). Supporting decision-making regarding fertility preservation in patients with cancer: An integrative review. Eur. J. Cancer Care.

[12] Martins da Silva S., Clarke H., Jansa Perez M., Mackie N.E., Barber T.J., Kirkman-Brown J.C. (2026). UK guidelines for the medical and laboratory procurement and use of sperm, egg and embryo donors. Hum. Fertil..

[13] Schuurman T., Zilver S., Samuels S., Schats W., Amant F., van Trommel N.V., Lok C. (2021). Fertility-sparing surgery in gynecologic cancer: A systematic review. Cancers.

[14] Marin L., Vural N., Oktay K.H. (2025). Advances to fertility preservation in patients with gynaecological cancers. Expert. Rev. Endocrinol. Metab..

[15] Rodolakis A., Scambia G., Planchamp F., Acien M., Di Spiezio Sardo A., Farrugia M., Grynberg M., Pakiz M., Pavlakis K., Vermeulen N. (2023). ESGO/ESHRE/ESGE guidelines for the fertility-sparing treatment of patients with endometrial carcinoma. Hum. Reprod. Open.

[16] Morice P., Scambia G., Abu-Rustum N.R., Acien M., Arena A., Brucker S., Cheong Y., Collinet P., Fanfani F., Filippi F. (2024). Fertility-sparing treatment and follow-up in patients with cervical cancer, ovarian cancer, and borderline ovarian tumours: Guidelines from ESGO, ESHRE, and ESGE. Lancet Oncol..

[17] Stefanovic K., Dotlic J., Pilic I., Milosevic B., Mihaljevic O., Beleslin A., Stefanović A. (2025). A decade of experience in diagnostic and conservative treatment of endometrial malignancy: Oncologic and obstetrical outcomes from a referral oncofertility centre. Diagnostics.

[18] Likic Ladjevic I., Dotlic J., Stefanovic K., Milosevic B., Beleslin A., Mihaljevic O., Bila J., Vukovic I., Radojevic M., Vilendecic Z. (2025). Fertility-sparing surgery for non-epithelial ovarian malignancies: Ten-year retrospective study of oncological and reproductive outcomes. Cancers.

[19] Likic Ladevic I., Nesic D., Stefanovic A., Kadija S., Vilendecic Z., Pilic I., Dotlic J., Radojevic M., Milosevic B., Stefanovic K. (2022). Oncologic and reproductive outcomes of fertility-sparing surgery for borderline ovarian tumours: First presentation of 10-year experience from a Serbian referral centre. Arch. Biol. Sci..

[20] Szubert M., Suzin J., Wierzbowski T., Kowalczyk-Amico K., Gajek A. (2021). Fertility-sparing surgery for early-stage cervical cancer: A systematic review. Cancers.

[21] Salman L., Covens A. (2024). Fertility preservation in cervical cancer-treatment strategies and indications. Curr. Oncol..

[22] Petrică L.-A., Deacu M., Cozaru G.C., Mitroi A.F., Bălţătescu G.I., Enciu M., Cojocaru O., Nicolau A.-A., Baz A.R., Șerbănescu L. (2025). Interplay Between Immune Microenvironment CD8^+^ Tumor-Infiltrating Lymphocytes and PDL-1 Expression as Prognostic Markers in Invasive Cervical Squamous Cell Carcinoma. Medicina.

[23] Cibula D., Raspollini M.R., Planchamp F., Centeno C., Chargari C., Felix A., Fischerova D., Jahnn-Kuch D., Joly F., Kohler C. (2023). ESGO/ESTRO/ESP guidelines for the management of patients with cervical cancer—Update 2023. Int. J. Gynecol. Cancer.

[24] Li X., Jiang Z., Lu J., Chen X., Ge H., Wu X., Li J. (2023). Neoadjuvant chemotherapy followed by radical trachelectomy versus upfront abdominal radical trachelectomy for patients with FIGO 2018 stage IB2 cervical cancer. Gynecol. Oncol..

[25] Ronsini C., Solazzo M.C., Bizzarri N., Ambrosio D., La Verde M., Torella M., Carotenuto R.M., Cobellis L., Colacurci N., De Franciscis P. (2022). Fertility-Sparing Treatment for Early-Stage Cervical Cancer ≥ 2 cm: A Problem with a Thousand Nuances-A Systematic Review of Oncological Outcomes. Ann. Surg. Oncol..

[26] Smith E.S., Moon A.S., O’Hanlon R., Leitao M.M., Sonoda Y., Abu-Rustum N.R., Mueller J.J. (2020). Radical trachelectomy for the treatment of early-stage cervical cancer: A systematic review. Obstet. Gynecol..

[27] Kohler C., Plaikner A., Siegler K., Hertel H., Hasenbein K., Petzel A., Schubert M., Blohmer J.-U., Böhmer G., Stolte C. (2024). Oncologic and Fertility Outcomes After Simple Trachelectomy in Women with Early Cervical Cancer. J. Minim Invasive Gynecol..

[28] Li X., Xia L., Li J., Chen X., Ju X., Wu X. (2020). Reproductive and obstetric outcomes after abdominal radical trachelectomy (ART) for patients with early-stage cervical cancers in Fudan, China. Gynecol. Oncol..

[29] Terzic M., Makhadiyeva D., Bila J., Andjic M., Dotlic J., Aimagambetova G., Sarria-Santamera A., Laganà A.S., Chiantera V., Vukovic I. (2023). Reproductive and Obstetric Outcomes after Fertility-Sparing Treatments for Cervical Cancer: Current Approach and Future Directions. J. Clin. Med..

[30] Casadio P., La Rosa M., Alletto A., Magnarelli G., Arena A., Fontana E., Fabbri M., Giovannico K., Virgilio A., Raimondo D. (2020). Fertility sparing treatment of endometrial cancer with and without initial infiltration of myometrium: A single centre experience. Cancers.

[31] Corzo C., Barrientos Santillan N., Westin S.N., Ramirez P.T. (2018). Updates on conservative management of endometrial cancer. J. Minim. Invasive Gynecol..

[32] Galant N., Krawczyk P., Monist M., Obara A., Gajek L., Grenda A., Nicos M., Kalinka E., Milanowski J. (2024). Molecular Classification of Endometrial Cancer and Its Impact on Therapy Selection. Int. J. Mol. Sci..

[33] Markowska A., Chudecka-Glaz A., Pitynski K., Baranowski W., Markowska J., Sawicki W. (2022). Endometrial Cancer Management in Young Women. Cancers.

[34] Uccella S., Zorzato P.C., Dababou S., Bosco M., Torella M., Braga A., Frigerio M., Gardella B., Cianci S., Lagana A.S. (2022). Conservative Management of Atypical Endometrial Hyperplasia and Early Endometrial Cancer in Childbearing Age Women. Medicina.

[35] Emons G., Grundker C. (2021). The role of gonadotropin-releasing hormone (GnRH) in endometrial cancer. Cells.

[36] Ogunbiyi M.O., Oxley S., Graham R., Olaitan A. (2024). The oncological and reproductive outcomes of fertility-preserving treatments for stage 1 grade 1 endometrial carcinoma: A systematic review and meta-analysis. J. Obstet. Gynaecol..

[37] Falcone F., Laurelli G., Losito S., Di Napoli M., Granata V., Greggi S. (2017). Fertility preserving treatment with hysteroscopic resection followed by progestin therapy in young women with early endometrial cancer. J. Gynecol. Oncol..

[38] Floyd J.L., Campbell S., Rauh-Hain J.A., Woodard T. (2021). Fertility preservation in women with early-stage gynecologic cancer: Optimizing oncologic and reproductive outcomes. Int. J. Gynecol. Cancer..

[39] Guo Y., Zong X., Li H., Qiao J. (2022). Analysis of IVF/ICSI outcomes in infertile women with early-stage endometrial cancer and atypical endometrial hyperplasia after conservative treatment. J. Assist. Reprod. Genet..

[40] Etrusco A., Lagana A.S., Chiantera V., Mikus M., Arsalan H.M., d’Amati A., Vitagliano A., Cicinelli E., Favilli A., D’Amato A. (2024). Reproductive and Oncologic Outcomes in Young Women with Stage IA and Grade 2 Endometrial Carcinoma Undergoing Fertility-Sparing Treatment: A Systematic Review. Biomolecules.

[41] Piatek S., Michalski W., Sobiczewski P., Bidzinski M., Szewczyk G. (2021). The results of different fertility-sparing treatment modalities and obstetric outcomes in patients with early endometrial cancer and atypical endometrial hyperplasia: Case series of 30 patients and systematic review. Eur. J. Obstet. Gynecol. Reprod. Biol..

[42] Mutlu L., Manavella D.D., Gullo G., McNamara B., Santin A.D., Patrizio P. (2022). Endometrial cancer in reproductive age: Fertility-sparing approach and reproductive outcomes. Cancers.

[43] Canlorbe G., Lecointre L., Chauvet P., Azaïs H., Fauvet R., Uzan C. (2020). Borderline ovarian tumours: CNGOF guidelines for clinical practice—Therapeutic management of early stages. Gynecol. Obstet. Fertil. Senol..

[44] Kim S.S. (2021). Fertility preservation for women with borderline ovarian tumours: Fertility-sparing surgery. Fertil. Steril..

[45] Cheung A., Shah S., Parker J., Soor P., Limbu A., Sheriff M., Boussios S. (2022). Non-epithelial ovarian cancers: How much do we really know?. Int. J. Environ. Res. Public Health.

[46] Sessa C., Schneider D.T., Planchamp F., Baust K., Braicu E.I., Concin N., Godzinski J., McCluggage W.G., Orbach D., Pautier P. (2020). ESGO-SIOPE guidelines for the management of adolescents and young adults with non-epithelial ovarian cancers. Lancet Oncol..

[47] Johansen G., Dahm-Kahler P., Staf C., Floter Radestad A., Rodriguez-Wallberg K.A. (2021). Reproductive and obstetrical outcomes with the overall survival of fertile-age women treated with fertility-sparing surgery for borderline ovarian tumours in Sweden: A prospective nationwide population-based study. Fertil. Steril..

[48] Kumari S., Kumar S., Bhatla N., Mathur S., Thulkar S., Kumar L. (2021). Oncologic and reproductive outcomes of borderline ovarian tumours in Indian population. Gynecol. Oncol. Rep..

[49] Sagnıc S., Karadag C., Tuncer H.A., Dogan S., Simsek T. (2023). Reproductive and oncologic outcomes in women with non-epithelial ovarian cancer: Single centre experience over 25 years. Turk. J. Obstet. Gynecol..

[50] Johansen G., Dahm-Kahler P., Staf C., Floter Radestad A., Rodriguez-Wallberg K.A. (2019). Fertility-sparing surgery for treatment of non-epithelial ovarian cancer: Oncological and reproductive outcomes in a prospective nationwide population-based cohort study. Gynecol. Oncol..

[51] Qi Y., Wang M., Yang Y., Zeng Z., Zhou Y. (2021). Analysis of factors influencing relapse and pregnancy in patients with borderline ovarian tumours. J. Cancer.

[52] Tunc M., Akıllı H., Gunakan E., Haberal A.N., Haberal A., Ayhan A. (2025). Borderline ovarian tumours: Recurrence patterns and management in a single-institution retrospective study. Taiwan J. Obstet. Gynecol..

[53] Lin X.X., Tang L., Yang M.J., Zhang B.L. (2024). Effects of fertility-sparing surgery on prognosis and fertility of patients with ovarian borderline tumours. Zhongguo Yi Xue Ke Xue Yuan Xue Bao.

[54] Wang R., Xiao Y., Fernando T., Chen Y., Zhu X. (2026). Oncofertility outcomes after fertility-sparing surgery for borderline ovarian tumours: Results of a large retrospective study. J. Ovarian Res..

[55] Cosyns S., De Vos M. (2025). Fertility following fertility-sparing surgery for borderline ovarian tumours. Semin. Reprod. Med..

[56] Canlorbe G., Chabbert-Buffet N., Uzan C. (2021). Fertility-sparing surgery for ovarian cancer. J. Clin. Med..

[57] Necula D., Istrate D., Mathis J. (2020). Fertility preservation in women with early ovarian cancer. Horm. Mol. Biol. Clin. Investig..

[58] Piatek S., Szymusik I., Sobiczewski P., Michalski W., Kowalska M., Ołtarzewski M., Bidzinski M. (2023). Obstetric results after fertility-sparing management of non-epithelial ovarian cancer. Cancers.

[59] Dolmans M.M., Hossay C., Nguyen T.Y.T., Poirot C. (2021). Fertility preservation: How to preserve ovarian function in children, adolescents and adults. J. Clin. Med..

[60] Arecco L., Blondeaux E., Bruzzone M., Ceppi M., Latocca M.M., Marrocco C., Boutros A., Spagnolo F., Razeti M.G., Favero D. (2022). Safety of fertility preservation techniques before and after anticancer treatments in young women with breast cancer: A systematic review and meta-analysis. Hum. Reprod..

[61] Practice Committee of the American Society for Reproductive Medicine (2020). Testing and interpreting measures of ovarian reserve: A committee opinion. Fertil. Steril..

[62] Purandare N.C., Ryan G.A., El Helali A., Crosby D. (2025). Fertility stimulation protocols in women with cancer. Int. J. Gynecol. Obstet..

[63] Tsampras N., Roberts S.A., Gould D., Fitzgerald C.T. (2018). Ovarian response to controlled ovarian stimulation for fertility preservation before oncology treatment: A retrospective cohort of 157 patients. Eur. J. Cancer Care.

[64] Wu M., Zhu Q., Huang Y., Tang W., Dai J., Guo Y., Xiong J., Zhang J., Zhou S., Fu F. (2023). Ovarian reserve in reproductive-aged patients with cancer before gonadotoxic treatment: A systematic review and meta-analysis. Hum. Reprod. Open.

[65] Dolinko A.V., Farland L.V., Missmer S.A., Srouji S.S., Racowsky C., Ginsburg E.S. (2018). Responses to fertility treatment among patients with cancer: A retrospective cohort study. Fertil. Res. Pract..

[66] Cioffi R., Pagliardini L., Quartucci A., Papaleo E., Vanni V.S., Provenzano S., Bertulli R., Candiani M., Mangili G. (2023). Response to controlled ovarian stimulation is not impaired in young patients with a sarcoma: Results from a monocentric case-control study. Cancers.

[67] McClam M., Xiao S. (2022). Preserving oocytes in oncofertility. Biol. Reprod..

[68] Hughes T., Harper A., Gupta S., Frazier A.L., van der Graaf W.T.A., Moreno F., Joseph A., Fidler-Benaoudia M.M. (2024). The current and future global burden of cancer among adolescents and young adults: A population-based study. Lancet Oncol..

[69] Gică N., Vișoiu I., Mocanu I.-C., Năstac A., Sima R.M., Panaitescu A.M., Mehedințu C. (2021). Fertility Preservation Strategies in Female Cancer Patients: Current Approaches and Future Directions. Medicina.

[70] Wnuk K., Switalski J., Miazga W., Tatara T., Religioni U., Olszewski P., Augustynowicz A. (2023). The Usage of Cryopreserved Reproductive Material in Cancer Patients Undergoing Fertility Preservation Procedures. Cancers.

[71] Salzler M., Saithna A., Cote M.P., Matzkin E., Rossi M.J. (2025). Lost but Not Forgotten: How to Manage Follow-Up Loss in Clinical Research. Arthroscopy.

[72] Al-Dorzi H.M., Arabi Y.M. (2024). Quality Indicators in Adult Critical Care Medicine. Glob. J. Qual. Saf. Healthc..

[73] Agha A.M., Aladul M.I. (2026). Healthcare Provider Perspectives on Barriers to Fertility Preservation in Oncology Care in Iraq: A Qualitative Study. Asia Pac. J. Clin. Oncol..

[74] Ng Q.J., Ee T.X., Goh C.S.Y., Lee S.H., Low J.E.H., Lin J., Phoon J.W.L., Chan J.J., Chin F.H.X. (2025). Oncofertility care and fertility preservation in gynaecological cancer: Experience from Singapore’s largest tertiary centre. Singap. Med. J..

[75] Tang T.W., Goh S.Y.C., Phoon J.W.L. (2025). A review of fertility preservation in women in Singapore from 2010 to 2019. Ann. Acad. Med. Singap..

[76] Debnath A., Achary T., Halder P., Dasgupta A., Mandal I., Charag S., Panwar N., Mondal A., Goswami B., Rao S. (2026). Time to treatment initiation delays in cervical cancer worldwide: A systematic review and meta-analysis. Cancer Epidemiol..

[77] Anderson A., Cooper J., Oyston C., Miles C., Sowden K., Peiris-John R., Wise M.R. (2022). Audit of waiting time-to-treatment of atypical endometrial hyperplasia. Aust. N. Z. J. Obstet. Gynaecol..

[78] ESHRE Guideline Female Fertility Preservation. https://www.eshre.eu/Guidelines-and-Legal/Guidelines/Female-fertility-preservation.

[79] Government of the Republic of Serbia. Healthcare in Serbia. https://welcometoserbia.gov.rs/healthcare-and-health-insurance.

[80] Serbian Society for Fertility Preservation and Oncofertility. https://www.onkofertilitetsrbija.rs/en/.

